# Immune-Driven Pathogenesis of Neurotoxicity after Exposure of Cancer Patients to Immune Checkpoint Inhibitors

**DOI:** 10.3390/ijms21165774

**Published:** 2020-08-11

**Authors:** Noelia Vilariño, Jordi Bruna, Foteini Kalofonou, Garifallia G. Anastopoulou, Andreas A. Argyriou

**Affiliations:** 1 Neuro-Oncology Unit, Hospital Universitari de Bellvitge-ICO L’Hospitalet, IDIBELL, 08908 Barcelona, Spain; nvilarino@iconcologia.net (N.V.); 35078jbe@comb.cat (J.B.); 2 Department of Oncology, Garry Weston Centre, Hammersmith Hospital, Imperial NHS Healthcare Trust, W120HS London, UK; kalfotini@gmail.com; 3 Department of Medicine-Oncology Unit, Saint Andrew’s General Hospital of Patras, 26335 Patras, Greece; liaanastopoulou@yahoo.gr; 4 Neurological Department, Saint Andrew’s General Hospital of Patras, 26335 Patras, Greece

**Keywords:** cancer, immunotherapy, immune checkpoint inhibitors, pathogenesis, immunopathology

## Abstract

Over the last decade, immune checkpoint inhibitors (ICIs) have revolutionized the treatment of several cancer types. ICIs work through the blockage of immune inhibitory signals, while increasing the T-cell specific immune antitumoral response. However, due to the fact that ICIs’ mechanism of action is not tissue antigen-specific and not limited to the tumor microenvironment, the use of cancer immunotherapy can produce a broad range of immune-related adverse events (irAEs). Neurological immune-related adverse events (NirAEs) are rare (the overall incidence varies between 1% to 6%), and these adverse events mainly concern the peripheral nervous system, rather than the central nervous system. Due to their potential severity, which could cause interruptions to cancer treatment, NirAEs are of particular clinical importance. Currently, the pathogenesis of these complications is not completely understood, although T-cells seem to play a principal role. Nevertheless, the development of NirAEs is likely to be a multifactorial and complex process. This conclusion can be extracted from the wide range of neurological auto-inflammatory and autoimmune disorders triggered or exacerbated by ICIs, and the extensive variability of the limited histological findings reported. The aim of this review is to summarize the potential immune-driven pathological mechanisms of NirAEs.

## 1. Introduction

Over the last decade, the blockade of the immune checkpoints with monoclonal antibodies (ΜAb) has significantly improved clinical outcomes in various malignancies. T-lymphocyte-associated protein 4 (CTLA-4) and programmed cell death protein 1 (PD-1) are receptors that, under physiological conditions, can help to maintain immune tolerance [1]. Immune checkpoint inhibitors (ICIs) enhance T-cell adaptive immunity against the tumor by targeting these receptors and improving overall clinical outcomes, either used as monotherapy or in combination with other conventional chemotherapeutic drugs. However, given the key roles played by CTLA-4 and PD-1/PD-L1 in immune system homeostasis, the blockage of this pathway has the potential to increase the relative risk of systemic immune-related overactivation and immune-mediated toxicities.

Anti-CTLA-4 treatment enhances naive T-cells mostly at early stages in the lymphoid organs, whereas anti-PD-1 antibodies induce antigen-mediated T-effector cells’ activation mainly in the peripheral tissues [2]. T-cell activation in the early stages of the immune cycle could explain why CTLA-4 antibodies induce more toxicity and fatal events compared with anti-PD-1 blockade [3,4] and why strategies of combined immune checkpoint blockade are associated with an increased frequency of immune-related adverse events (irAEs) [5,6]. A recent meta-analysis did not show an increase in the incidence of neurological immune-related adverse events (NirAEs) when CTLA-4 inhibitors were combined with PD-1 inhibitors, as opposed to ICI monotherapy, which is related to multiorgan irAEs [7].

The most common irAEs involve the skin, gastrointestinal tract, endocrine system and liver and can occur in up to 65% of ICI-exposed patients [8]. NirAEs occur less frequently in an estimated 1–6% of patients treated with ICIs. NirAEs involve a wide spectrum of clinical and pathological disorders of both the central and peripheral nervous systems [9,10]. Despite this comparatively lower frequency with regard to other organs, the incidence of NirAEs is associated with a three- to four-fold higher incidence rate than other primary neurological autoimmune diseases, such as acute inflammatory demyelinating neuropathies, myasthenia gravis or multiple sclerosis (https://www.orpha.net/consor/cgi-bin/index.php) [8].

The reasons behind the low incidence of immune-related toxicity in the nervous system compared to other organs remain largely unknown. Typically, the CNS and the peripheral nervous system (PNS) have been considered as immune-privileged systems due to the presence of anatomical barriers such as the blood–brain or the blood–nerve barrier [11,12], as well as peculiarities in the population of resident cells within the tumor microenvironment, with the example of microglia and astrocytes. Additionally, the lymphatic system structure differentiates the nervous system from the other organs and can play an important role in the incident rates of NirAEs [13,14]. The above factors, together with probably other, still unknown elements of immune activation might be essential in the formation of a unique environment to differentiate the immune system from the other peripheral organs. Furthermore, an unresolved question that distinguishes the CNS from the other peripheral organs is the ability of ICIs to cross the blood–brain barrier. Currently, the exact mechanism of action of ICIs in the brain remains unknown. ICIs either work locally or as a consequence of the transmigration of the immune system upon activation, which has potential implications in the development of NirAEs.

## 2. Clinical Phenotype of NirAEs

ICIs can both attack the peripheral and central nervous system (CNS), although neuromuscular involvement is more frequently disclosed than CNS involvement (5.5% and 0.46%, respectively) [7]. The most commonly encountered CNS clinical syndromes include meningitis, encephalitis, vasculitis, myelitis and cranial neuropathies, occurring as a result of neuroinflammation. Overall, data from anti-PD1/PD-L1 monotherapy studies (125 clinical trials; 20,128 patients) showed a very low incidence of CNS-related adverse events, estimated as being lower than 0.5% after exposure to pembrolizumab, nivolumab and ipilimumab therapy [15]. Notably, based on the results of a Japanese pharmacovigilance database, it was evident that meningitis, encephalitis and myelitis were more significantly associated with the PD-L1 agent nivolumab [16]. In any case, ICI-related encephalitis might be a serious (grade 3) adverse event with a relatively high mortality rate [16,17]. On the other hand, CNS vasculitis, taking the form of giant cell arteritis and isolated retinal vasculitis, has a much more benign natural course. Finally, the exacerbation of known multiple sclerosis or de novo manifestation of CNS demyelination has also been reported after exposure to ICIs. Notably, de novo CNS demyelination has been associated with enhanced responses of myelin-reactive peripheral CD4+ T-cells [16,17]. Nonetheless, data are currently scarce and one cannot, with confidence, conclude as to whether ICI treatment is appropriate in cancer patients with pre-existing CNS inflammatory disease [17]. This issue will be discussed in more depth in a subsequent section, i.e., 3.5.

On the other hand, myositis appears to be the most common neuromuscular syndrome, with diffuse myalgias in back and proximal limbs being clinically evident in up to 2.95% of patients exposed to anti-PD-1 and PD-L1 [15]. Data from anti-PD-1 monotherapy studies (3336 patients with nivolumab and 3301 patients with pembrolizumab) are in keeping with a greater incidence of myositis (2.6% for nivolumab and 1.07% for pembrolizumab) than of peripheral neuropathy (0.73% and 0.28%, respectively) [9]. Moreover, ICI combinations are also associated with a greater risk of myopathies than peripheral neuropathy manifestation [9]. Notably, a meta-analysis focusing on the severe toxic effects associated with ICIs found a significantly increased incidence of myositis, when anti-CTLA4 and anti-PD1 or PD-L1 were administered in combination [4]. Contrary to the results of the latter study, another meta-analysis dealing with high-grade adverse events failed to document a higher risk of developing myositis when ICIs were given either as monotherapy or combined [7].

Conversely, about 1% of patients exposed to therapy with PD-1/PD-L1 inhibitors will manifest any grade of peripheral nerve damage in the form of axonal sensory peripheral neuropathy. The incidence of peripheral neuropathy is comparable after therapy with anti PD-1/PD-L1 or CLTA4 drugs (0.73% vs. 0.79%, respectively). Treatment-emergent grade 3 neurotoxicity is much less likely to occur with PD-1/PDL-1 therapy than with conventional chemotherapy agents [7,10,15].

## 3. Mechanisms of NirAE Pathogenesis

**Figure 1:**
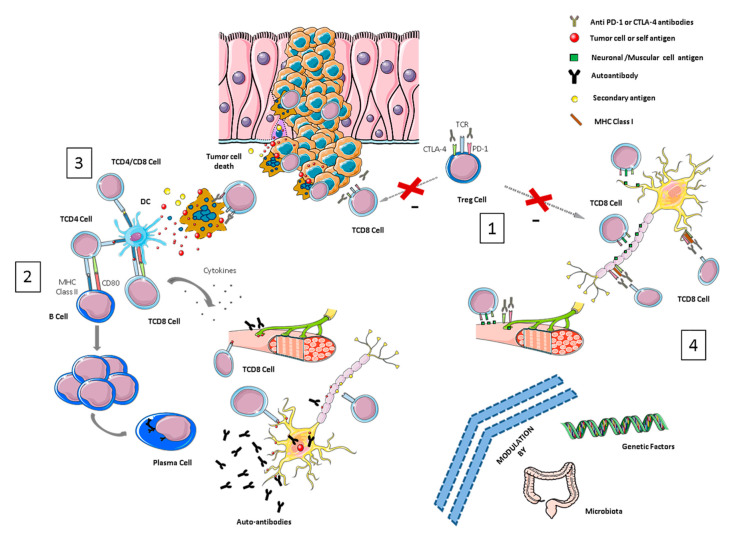
Overview of pathological mechanisms underlying neurological immune-related toxicities (NirAEs). (1) Regulatory T-cells (Tregs) are key players in maintaining immune tolerance and tumor immune-suppression within the tumor microenvironment by suppressing effector T-cells. CTLA-4 and PD-1 are receptors expressed by these cells. Treg cell blockade by immune checkpoint inhibitors (ICIs) may induce Treg reduction and the disruption of CD8 + T-cell control, resulting in the proliferation and activation of effector cells. Thus, an immune toxicity development can be started through the recognition of self-tissue antigens, such as neuronal or muscular antigens. (2) The molecular similarity between a tumor-antigen and a self-antigen recognized by effector cells might induce T- or B-autoreactive cells wrongly directed against self-antigens in a process called molecular mimicry, inducing autoimmune reactions against normal tissues. (3) Cytotoxic CD8+ T-cells, after recognizing tumor antigens, induce cytokine secretion and the cytotoxic death of tumor cells. This process can also wrongly produce the death of non-transformed bystander cells releasing secondary antigens. The primary and secondary antigens released by both types of cells (antigen spreading) might be ingested and processed by antigen-presenting cells (APCs). These activated APCs can prime new T- or B-cells that can cause additional tumor and normal tissue destruction, leading to an autoimmunity process. (4) ICI antibodies could recognize their target molecules, which are also expressed by non-hematopoietic cells including those of the nervous system (such as astrocytes and neurons) and thereby directly induce local injury through antibodies or T-cell cytotoxicity mechanisms.

Several mechanisms have been proposed to be implicated in the development of irAEs, such as T-reg down-regulation, the cross-presentation of neoantigens, epitope spreading, genetic predisposition and microbiome alteration, yet the pathogenesis behind ICI-related toxicities remains largely unknown [18,19]. The complexity of the immune system, together with the relatively low incidence of NirAEs, and the limited access to histological samples (especially in the case of CNS tissue), impairs our understanding of the biological process involved in the development of these autoimmune toxicities. In this review, we describe the potential molecular mechanisms underlying the development of different neurological toxicities after immunotherapy (see Figure 1).

### 3.1. Breaking Immune Tolerance

The mechanism of ICIs’ action is not tissue antigen-specific, and their targets, the immune checkpoint receptors (CTLA-4, PD-1 and others), are not only expressed on T-cells. Other immune cells and tissues might also be affected by ICI blockade. Regulatory T-cells (Tregs) express most of the checkpoint molecules, including CTLA-4 and PD-1, and represent a direct target of ICI immunotherapy. Tregs are key players in maintaining peripheral tolerance by actively suppressing effector T-cells [20] and by inducing, at the same time, immune suppression within the tumor microenvironment [21]. Experimental evidence has shown that anti-CTLA-4 ΜAbs’(Monoclonal Antibodies) efficacy depends on the depletion of these Treg cells within the tumor microenvironment [22]. At the same time, studies of Tregs in autoimmune neurological disorders, such as multiple sclerosis and experimental autoimmune encephalomyelitis, reveal critical roles for these cells in the prevention and reduction of neuro-inflammation [23]. It is possible that the development of NirAEs can be partly attributed to the systemic depletion of Tregs, which, in turn, might result in the loss of immune tolerance [19].

On the other hand, CTLA-4 and PD-1 are also key players in the activation, proliferation and regulatory function of B-cells [24,25]. A clinical study has shown that CTLA-4 inhibition may lead to a loss of B-cell self-tolerance, by inducing the development of common serum autoantibodies. In a cohort of 199 melanoma patients, seroconversion was detected in 19.2% (19 of 99) after ipilimumab treatment. However, in this study, the authors observed a non-significant association between the development of autoantibodies and any irAEs (odds ratio, 2.92; 95% CI, 0.85–10.01; *p* = 0.12), but no NirAEs were reported, despite the high odds ratio observed. The wide range of the CI due to the small number of patients with positive autoantibodies (*n* = 19) most probably accounts for the latter insignificant association [26].

In addition, immune homeostasis is orchestrated by the interactions between immune cells and the cytokine environment. Pro-inflammatory cytokines cause the destruction of target tissues and a break of tolerance, whereas anti-inflammatory cytokines maintain immune tolerance. Tregs are highly regulated by a vast and intricate milieu of cytokine signals, such as IL-2 or TGF-β, which ultimately might modulate immune tolerance [27]. ICIs have shown distinct effects on the plasma levels of cytokines [28], and these molecules may also be involved in the pathophysiology of irAEs [17]. Cytokines are important regulators of host immune activity and may serve as potential prognostic or predictive biomarkers in patients treated with ICIs. Recently, baseline serum IL-8 levels were associated with poor outcomes in two large retrospective cohorts of patients treated with ICIs [29,30], and elevated baseline IL-17 levels were a predictive marker of immune-related colitis in melanoma patients treated with ipilimumab [31]. Moreover, Lim et al., using the 65-plex Human Cytokine/Chemokine Discovery Assay, reported that the elevated expression of 11 circulating cytokines (G-CSF, GM-CSF, fractalkine, FGF-2, IFNa2, IL12p70, IL1a, IL1B, IL1RA, IL2 and IL13) correlates with severe irAE development. The integration of these 11 cytokines into a single toxicity score (CYTOX) predicted irAEs in an independent cohort of patients with melanoma treated with an ICI combination. These cytokines have pro-inflammatory activities (immune cell recruitment, proliferation, survival, differentiation and effector functions), and many of them have been also implicated in the inflammation that underlies autoimmune diseases [32]. To our knowledge, none of these cytokines is significantly related with NirAEs.

Further investigation and research are needed to elucidate the exact mechanisms behind the occurrence of NirAEs. The blockade of different subtypes of immune cells—i.e., T-cells, B-cells or Tregs—together with systemic changes in cytokines leading to the loss of immune system homeostasis can trigger an innate immune response and the activation of self-reactive immune cells. Host factors, such as genetic predisposition (for example, HLA genes, which play a central role in antigen presentation and immune tolerance) or the microbiome composition (microbiome-derived products), can further augment this immune response overactivation. The latter theory might explain the reasons behind potential differences in the spectrum and frequency of irAEs.

### 3.2. Molecular Mimicry and Cross-Reactivity Among Tumor- and Self-Tissue Antigens

The concept of molecular mimicry refers to the similarity between a host antigen and an antigen from a microorganism, environmental agent or even a tumor cell. This molecular likeness might induce a T-cell or autoantibody cross-reactive autoimmune response wrongly directed against the host antigen rather than the pathogenic antigen. However, this a complex process in which host genetic and environmental factors, as well as issues related to the mechanisms associated with a positive/negative selection of T- and B-cells, are also involved. In patients treated with ICIs, there is increasing evidence that a phenomenon of the cross-presentation of neo-antigens or shared antigens (between tumor cells and normal host cells) might induce a loss of tolerance and subsequent autoimmune reaction. The self-antigens might be released when tumor or non-tumor tissues, distributed in and around the tumor environment, are damaged collaterally, by immune cells directed against the tumor [18,33].

A classic and well-known example of a cross-reactive immune response against a self-antigen is paraneoplastic syndrome (PNS) [34,35]. Some authors have suggested that ICIs could promote immune-mediated paraneoplastic syndromes (PNSs) via a cross-reactive immune response against a self-antigen expressed on both neural cells and tumor cells [36]. This idea is sustained by the clinical findings in PNS studies [34,35] and supported by preclinical findings in which CTLA-4 blockade elicited paraneoplastic neurological disease in a mouse model, after the induction of neo-self antigen expression, both in the cerebellar Purkinje neurons and an implanted breast tumor [37].

Likewise, a case of anti-N-methyl-D-aspartate receptor (NMDAR) autoimmune encephalitis was reported in one patient with melanoma. The presence of NMDAR antibodies in the blood and cerebrospinal fluid supports immune-related pathogenesis. NMDAR is expressed on melanocytes, and *GRIN2A* gene, which encodes the NMDAR subunit GluN2A, is highly mutated in melanoma patients. This case may represent a molecular mimicry phenomenon in which antibodies against NMDAR attack both melanoma and CNS cells [38,39].

This mechanism of molecular mimicry has also been suggested for some melanoma patients who developed ICI-related demyelinating polyneuropathy, since melanocytes and Schwann cells originate from the neural crest and share many epitopes for humoral and cellular immune responses [40]. In further support of this concept, two cases of fatal myocarditis have been reported, in which similar T-cell clones were found in the myocardium, skeletal muscle and tumor deriving from the same patient. This finding suggests that a common antigen shared between the normal tissue (myocardium and muscle) and tumor was recognized by the same T-cell clones [41].

Overall, ICIs may unmask or accelerate pre-existing autoimmune reactions against neuronal antigens, leading to the development of NirAEs. Therefore, patients with cancer types that are frequently associated with PNS neurotoxicity, such as melanoma and small cell lung cancer, might be at an increased risk of developing neuromuscular NirAEs when treated with ICIs. In line with the latter view, a retrospective study reported a significant increase in the frequency of anti-Ma2 encephalitis following the implementation of ICIs [42]. Surprisingly, to our knowledge, an increased incidence of PNSs was not observed among the phase III clinical trials of ICIs for those tumors more frequently associated with PNPs (Peripheral Neuropathies) [6]. However, the low incidence of these complications or the inherent bias in the study cohorts and clinical series may jeopardize these deductions.

In contrast to primary neurological autoimmune diseases, in almost all of the ICI-related inflammatory demyelinating neuropathies reported, antiganglioside antibodies were not found and excellent responses to steroid therapy were observed [10]. Moreover, the worst refractory outcomes were observed in myasthenic syndromes, despite the usually intensive therapy employed [43], indicating overall multifactorial involvement in addition to the cross-reactivity process.

### 3.3. Epitope Spreading

Some authors have suggested that irAEs may be caused by immunotherapy-induced epitope spreading (ES) [18]. Tissue damage, during the response to immunotherapy, may lead to the release of secondary tumor and non-tumor antigens that prime subsequent immune responses. ES, in contraposition to cross-reacting antibodies, is the expansion of an immune response due to secondary antigens (distinct from the primary one) initially not recognized by the original effector T-cell. These antigens are processed and presented by antigen-presenting cells to prime B- and T-cells that can access the tissue, and they cause immune-mediated responses. This process has been reported in patients receiving different types of immunotherapy (tumor vaccines, adoptive transfer of CTLs and Chimeric Antigen Receptor T cells—CAR T cells). Some preclinical and clinical evidence suggests that therapy with ipilimumab and PD-1/PD-L1 inhibitors can also induce epitope spreading [44,45]. Therefore, it is likely that tumor-specific immune activation by epitope spreading can trigger autoimmunity against normal self-tissues, leading to irAE development.

Certain chemotherapeutic drugs or radiotherapy can lead to immunogenic cell death through tumor-antigen processing and presentation to the immune system [46]. Recently, ICIs and chemotherapy or radiotherapy combinations have become the new standard of care against solid tumors, such as non-small cell lung cancer (NSCLC). In this context, despite the lack of direct comparisons, phase III clinical trials of chemotherapy plus pembrolizumab do not seem to show an increased incidence of irAEs compared to that with pembrolizumab monotherapy (22.7% and 28.8% vs. 29.2% and 28%, respectively), and this also applies for NirAEs [47,48,49,50]. Therefore, although ES can contribute to the irAE mechanism, there is lack of preclinical and clinical evidence to support its contribution to the presentation of NirAEs.

### 3.4. Recognition of Target Molecules (CTLA-4 and/or PD-1/PD-L1) in Nervous System Tissues

IrAEs can also be derived from the “off-target” effects of ICIs on non-hematopoietic cell lines bearing the target immune checkpoint ligand. In support of this mechanism, it was identified that CTLA-4 protein expression on pituitary endocrine cells might be directly targeted by the anti-CTLA4 MAb [51]. Caturegli et al. confirmed the expression of CTLA-4 in pituitary cells in an autopsy series of patients who received anti-CTLA-4 antibodies and a higher level of pituitary CTLA-4 expression in a patient with clinical and histological evidence of severe hypophysitis also associated with T-cell infiltration and IgG-dependent complement fixation and phagocytosis. The authors suggested that this toxicity likely occurred owing to a combination of mechanisms, including antibody-dependent complement-mediated cytotoxicity and also direct T-cell-mediated cytotoxicity [52]. Moreover, hypohysitis was also reported, at a lower frequency, in patients treated with PD-1 MAb, suggesting that not only the expression of CTLA-4 in the pituitary gland could explain this toxicity.

Immune checkpoint receptors such as CTLA-4, as mentioned above, are distinctly expressed in different tissue microenvironments, providing a means for selective roles in tissue tolerance. PD-1 and its ligands can also be expressed on a wide variety of non-hematopoietic cell types, including vascular endothelial cells, fibroblastic reticular cells, epithelial cells, pancreatic islet cells, astrocytes and neurons [20,36]. Specifically, PD-1 protein expression was observed in the cortex and basal ganglia, and PD-1, PD-2 and CTLA-4 RNA expression was verified over the whole CNS, although CTLA-4 is preferentially expressed in the brainstem and spinal cord (https://www.proteinatlas.org/). Temporal as well as spatial differences in immune checkpoint expression may contribute to distinct immune-regulatory functions and differences in toxicities, but further studies are needed.

### 3.5. Exacerbation of Previous Autoimmune Reactions

Blocking the interaction between PD-1/PD-L1 or CTLA-4 on lymphocytes resident in or infiltrating the nervous system could increase local inflammation or reveal latent central or peripheral inflammation. This mechanism might be responsible for the exacerbation of multiple sclerosis, autoimmune encephalitis, inflammatory demyelinating neuropathies or PNSs, as shown, for example, in some experimental models of CNS inflammation [36,53,54].

ICIs might augment pre-existing immune responses directed against the nervous system. Several case reports have described the exacerbation of neurological autoimmune diseases during or after ICI therapy. A transition from radiologically isolated syndrome to clinically definite multiple sclerosis in a patient with metastatic melanoma after ipilimumab treatment has been reported. Using TCR gene sequencing analysis, the authors compared the TCR repertoire of T-cells present in the primary melanoma with the TCR repertoire in two consecutive cerebrospinal fluid samples. The analysis revealed two distinct oligoclonal CD4+ and CD8+ T-cell repertoires induced by ipilimumab: an immune response against the tumor and one against neural antigens, which resulted in severe multiple sclerosis disease activity [55].

In addition, the exacerbation of peripheral neurological autoimmune diseases such as pre-existing myasthenia gravis in patients under ICI blockade has also been highlighted in some clinical reports [56,57]. However, a systematic review of observational studies, case reports and series of patients receiving ICI for the treatment of cancer, who also happened to have a pre-existing autoimmune disease, did not show any differences in the proportion of irAEs between patients with active and those with inactive autoimmune diseases. Moreover, both groups presented a probability of around 50% of suffering an exacerbation of their autoimmune disease [58]. Unfortunately, these proportions cannot be compared with those in the trial patient population, due to unreported grades of severity concerning the irAEs. Despite several clinical case reports, a more solid clinical response is difficult to achieve, since patients with autoimmune diseases have been excluded from clinical trials involving ICIs, due to their potential higher risk of serious irAEs.

### 3.6. Genetic and Microbiome-Related Factors

The reason accounting for why some patients develop irAEs while others do not remains unclear. Currently, a relationship between some genetic factors and the risk of irAEs has not been established. For example, in terms of genetic associations, CTLA-4 polymorphisms have been linked to an increased risk of autoimmune diseases, such as type 1 diabetes, and preclinical models have shown that anti-CTLA-4 can increase the risk of autoimmune diabetes [59]. However, a recent single nucleotide polymorphism analysis of high-risk loci for type 1 diabetes in a human case of fulminant type 1 diabetes treated with an ICI failed to reveal a high-risk genetic profile [60]. Likewise, in a pooled study involving 453 patients with melanoma who were treated with ipilimumab, no association was found between one specific genotype (HLA-A status) and the risk of irAEs [61]. In summary, the role of genetic predisposition is unclear with regard to identifying those patients who are at higher risk of develop irAEs during ICI treatment.

In addition to genetic factors, another relevant question concerns whether the microbiological composition of a patient’s gastrointestinal flora is related to the development of irAEs. In fact, the most common irAEs involve organs that are rich in commensal organisms, such as the skin, colon and lungs [62]. In a study of patients with metastatic melanoma undergoing ipilimumab treatment, *Bacteroidetes phylum* was correlated with resistance to the development of immune-related colitis [63].

However, further research with prospective studies is needed to determine whether genetic or microbiome compositions are augmenting or reducing the risk of irAEs. To our knowledge, there is not any evidence correlating genetic or microbiome-related factors, specifically when it comes to the development of NirAEs.

## 4. Clinical Implications in NirAE Management

The molecular pathogenesis of NirAEs remains largely unknown. As a result, the optimal management of NirAEs has not yet been established. The treatment of these adverse events involves the prompt discontinuation of ICI therapy upon clinical suspicion of NirAE evidence and the initiation of immunosuppressive therapy. As for the other irAEs, their optimal management requires the initial exclusion of tumor-related pathology (spinal cord compression or cerebral metastases) and the involvement of experienced multidisciplinary teams to guide appropriate investigations and tailor treatment strategies. Currently, administering corticosteroids represents the cornerstone of NirAEs’ management. Intravenous immunoglobulin, plasma exchange and other immune-modulating treatments should be considered in more severe cases [64]. However, with the aim of limiting the potential adverse effects of immunosuppression on the tumor response, depending on the most predominant immune infiltrate type, the use of new biological and non-biological immunosuppressive drugs—including tocilizumab, natalizumab and rituximab—might be rational. Toward the latter view, further prospective investigations on new therapeutic perspectives for managing refractory immune checkpoint-related toxicities, including NirAEs, are warranted to elucidate which is the optimal treatment approach to adhere to in severe and steroid-refractory immune-related adverse events [65].

## 5. Conclusions

Due to the key role of ICIs priming specific antitumor T-cell responses and the predominance of T-cell infiltration within histological samples of irAEs case reports, T-cells are believed to play a pivotal role in ICI-mediated toxicity. However, based on the wide range of neurological auto-inflammatory and autoimmune disorders observed in patients undergoing ICI treatment, other potential players are most likely involved in this process. A unique clinical picture, such as encephalitis, may be yielded by different mechanisms (autoantibodies, T-cell infiltration, etc.), and, at the same time, extrapolations made from the pathological mechanisms behind primary autoimmune diseases may not be representative of irAEs. Moreover, the functional roles of cytokines and innate immunity are yet to be extensively explored, while in the few clinical cases reported, their involvement in the development of irAEs can be highly suspected.

Unfortunately, the role of the primary tumor histology and the potential correlations with the type of ICI used are not well controlled for in large clinical trials and meta-analyses published with regard to the development of NirAEs, which could undoubtedly provide essential information on the underlying mechanisms of immune-related toxicities. Similarly, the concurrence of other irAEs in addition to NirAEs might also be a useful tool, in order to improve our knowledge about the mechanisms involved in the development of these adverse events and the role of host tissues.

Overall, as derived from the immune-driven hypothesis and clinical clues available to date, it is suggested that NirAEs might develop as a result of different mechanisms, while the stochastic emergence suggests potential genetic and microbiome-related factors additionally modulating these mechanisms. Such complexity highlights the need to increase our efforts to obtain clinical data, tissue samples and more detailed histological and molecular analysis, in order to enhance our depth of knowledge regarding the molecular pathogenesis of NirAEs. Reliable biomarkers are urgently needed, in order to identify patients that are at higher risk of developing toxicities and in order to improve the therapeutic management of and the clinical approach against irAEs, ensuring the least possible interference with the antitumoral effects of ICIs.

## Abbreviations

CTLA-4	T-lymphocyte-associated protein 4
PD-1	Programmed cell death protein 1
ICIs	Immune checkpoint inhibitors
irAEs	Immune related adverse events
NirAEs	Neurological immune-related adverse events
CNS	Central nervous system
Tregs	Regulatory T-cells
APCs	Antigen-presenting cells
PNS	Paraneoplastic syndrome
NMDAR	N-methyl-D-aspartate receptor
ES	Epitope spreading
NSCLC	Non-small cell lung cancer
Mab	Monoclonal antibodies
